# Effectiveness of a Progressive Rehabilitation Approach Without Sport Activity Restriction for Acute Lumbar Spondylolysis in High-Level Athletes: A Retrospective Case Series

**DOI:** 10.7759/cureus.103041

**Published:** 2026-02-05

**Authors:** Kanta Matsuzawa, Tatsuya Takahashi, Jun Sakata, Tomoya Uchida, Tatsuhiro Suzuki, Tadahiro Sakai

**Affiliations:** 1 Toyota Athlete Support Center, Toyota Memorial Hospital, Toyota, JPN; 2 Orthopedics, Toyota Memorial Hospital, Toyota, JPN

**Keywords:** conservative treatment, lumbar spondylolysis, physical therapy, return to sport, spine

## Abstract

Objective: This study aimed to examine the characteristics and clinical outcomes of high-level athletes with acute lumbar spondylolysis (ALS) treated with a progressive rehabilitation (PR) approach without rigid bracing or activity restriction.

Methods: This retrospective consecutive case series included seven high school or collegiate athletes competing at the national level who underwent a PR approach for ALS at our institution between January 2023 and December 2024. One athlete was excluded due to loss to follow-up, leaving six athletes for analysis. The intervention consisted of a PR program without rigid bracing or activity restriction, emphasizing stepwise mobility, stability, strengthening, and pain-based progression of functional movements. Main outcomes included MRI findings, pain status, return-to-sport (RTS) rate and period, and follow-up duration.

Results: Traumatic episodes were the most common etiological factor (66.7%), involving high ground reaction force movements or excessive lateral bending. MRI improvement was observed in five patients (83.3%), and pain resolution occurred in all six patients (100%). The RTS rate was 100%, with a median RTS period of 65 days (range, 54-112), which was shorter than previously reported for conservative treatment. No recurrence occurred during follow-up (median, 109 days).

Conclusions: A PR approach without rigid bracing or activity restriction enabled early RTS in high-level athletes with ALS, with symptom improvement and no recurrence. This approach may allow modification of pain-provoking or injury-related movements and help minimize declines in physical fitness and body composition associated with activity restriction. It may be suitable for post-growth high-level athletes who understand the risks related to bone healing and require timely RTS, although further research is needed to clarify stage-specific indications and long-term outcomes.

## Introduction

Lumbar spondylolysis is a stress fracture that occurs in the pars interarticularis of the lumbar spine [1, 2], whereas spondylolisthesis refers to a condition in which spondylolysis has progressed, resulting in anterior slippage of the vertebra. Based on imaging findings, lumbar spondylolysis is classified into very early, early, progressive, and terminal stages [3, 4]. Among these, cases showing bone marrow edema (BME) on short tau inversion recovery (STIR) magnetic resonance imaging (MRI) in the very early, early, or progressive stages are defined as acute lumbar spondylolysis (ALS) [5, 6].

Conservative treatment is generally the first-line approach for ALS [7, 8]. Traditional conservative treatment typically consists of rigid bracing and activity restriction until bony union is achieved, combined with physical therapy, such as stretching and strengthening exercises [7, 8]. Return to sport (RTS) is gradually initiated after bone healing is confirmed by follow-up imaging [8]. While ALS is most common among adolescent athletes during the growth period [2], several reports have described its occurrence in skeletally mature high-level athletes [5, 6, 9], presumably due to the high-intensity training demands in this population [5]. Reported RTS rates with traditional conservative treatment in high-level athletes include 100% [5], 90.9% [6], and 28.5% [9], indicating generally favorable but variable outcomes. However, Tezuka et al. [6] noted that pain resolution may be prioritized over bony union in high-level athletes, suggesting that conventional conservative treatment may not always be optimal. Therefore, it is necessary to establish a flexible treatment framework tailored to the needs of high-level athletes with ALS.

A major limitation of traditional conservative treatments is the prolonged time required to achieve RTS. To the best of our knowledge, the shortest reported RTS period for high-level athletes under conventional protocols is still about 150 days [6]. Activity restriction has been reported to reduce physical capacities such as endurance, speed, and power [10-13] and to induce unfavorable changes in body composition, including muscle atrophy [14, 15]. Based on these findings, it is reasonable to speculate that conventional conservative treatment for ALS, which typically involves activity restriction, may have similar effects and thereby potentially prolong the time required for RTS. In clinical practice, high-level athletes often find activity restriction challenging due to career considerations or psychological resistance. We encountered high school and collegiate athletes, all of whom were national-level competitors, for whom conventional conservative treatment was not feasible, because the athletes expressed concern that prolonged activity restriction would significantly interfere with their competition schedules and athletic development. Therefore, we implemented a progressive rehabilitation (PR) approach, omitting rigid bracing and activity restriction from the outset and initiating physical therapy that included functional movements, which yielded favorable clinical outcomes.

Therefore, the purpose of this study was to retrospectively examine the patient characteristics and clinical outcomes of high-level high school and collegiate athletes with ALS who underwent the PR approach and to exploratorily discuss its clinical usefulness and indications.

## Materials and methods

Design and patients

This retrospective consecutive case series identified all high school or collegiate athletes competing at the national level who underwent a PR approach for ALS at our institution between January 2023 and December 2024. During this period, seven eligible athletes were screened. Of these, one athlete was excluded due to loss to follow-up, leaving six athletes for the final analysis. ALS was defined based on MRI findings, specifically the presence of BME at the pars interarticularis, with terminal-stage lesions excluded, rather than symptom duration. The chief complaint in all athletes was lower back pain, diagnosed by MRI. In this study, low back pain was defined as athlete-reported pain localized to the lumbar region that was provoked by sport-specific or daily activities and clinically attributed to pars interarticularis pathology. Recent studies have reported that combining bone-like image MRI and STIR-MRI is useful for diagnosing lumbar spondylolysis [16]. Diagnosis was based on the presence of BME in the pars interarticularis on STIR-MRI or a fracture line on bone-like image MRI. This study was conducted in accordance with the Declaration of Helsinki and approved by the ethics committee. Informed consent for participation and publication was obtained from all participants and guardians if under 18 years.

Data collection

All patients were high-level athletes who had participated in national-level competitions. Demographic data (age and sex), sports, lesion level, and laterality were recorded. According to previous studies [17, 18], lesion stage was classified as very early, early, or progressive. Based on previous studies [6], etiological factors were classified into four categories: contralateral terminal-stage lesions, traumatic episodes, excessive exercise, and alternation in the athletic event. For athletes classified as having a traumatic episode, injury-related movements were recorded and further categorized according to similar movement patterns. Pain-provoking sport-specific movements were also documented for each case.

Progressive rehabilitation approach

**Figure 1:**
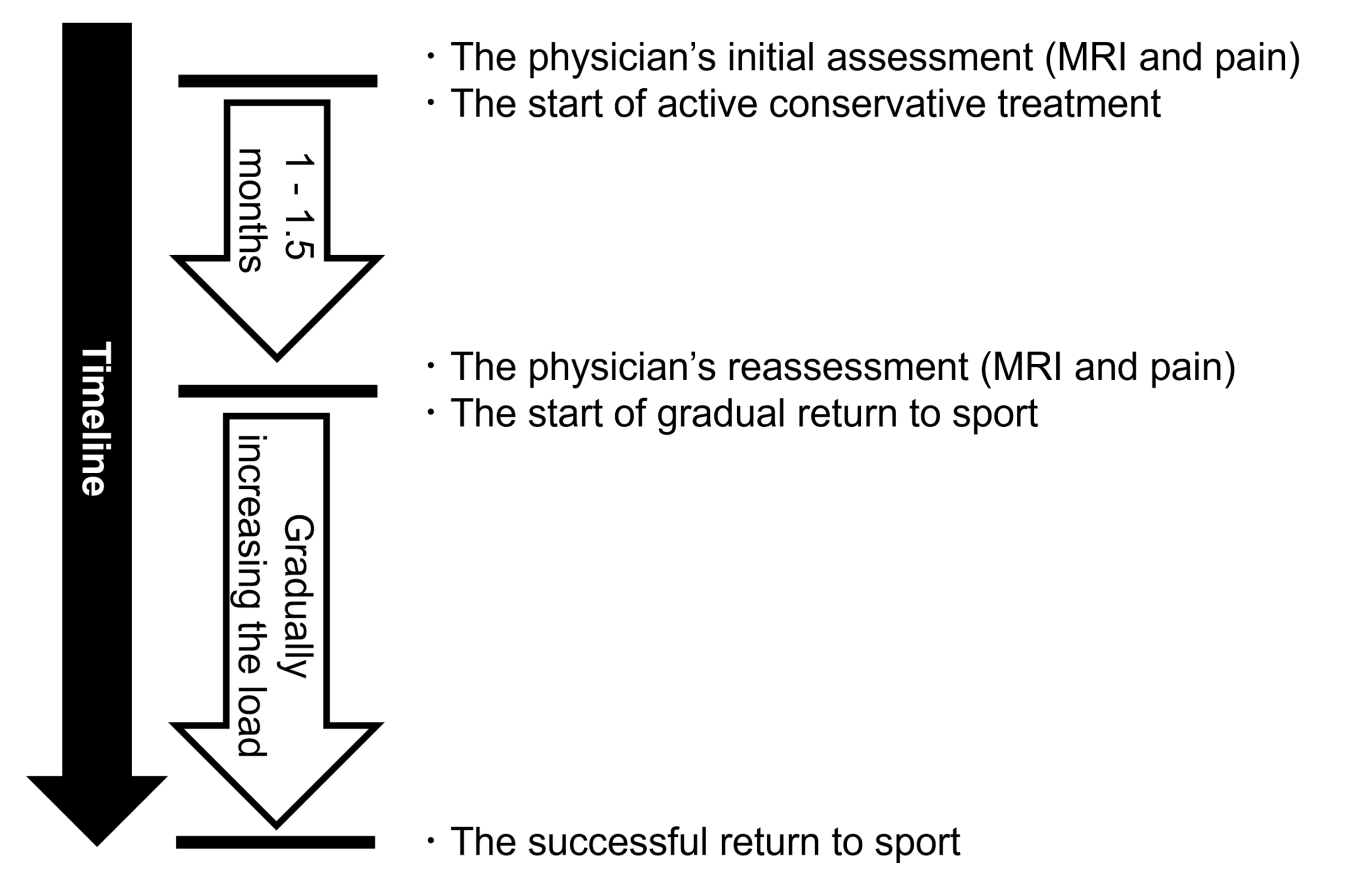
The protocol for the progressive rehabilitation approach. MRI, magnetic resonance imaging.

The protocol is illustrated in Figure 1. Initially, the physicians, who were orthopedic surgeons with more than 10 years of clinical experience, assessed both the MRI findings and pain symptoms. MRI was used to evaluate the presence or absence of a fracture line and BME, and pain was assessed during trunk flexion, extension, rotation, lateral bending, and the Kemp test [19]. No rigid bracing was applied from the initial visit, and activity restriction was generally not implemented; instead, physical therapy based on the PR approach was performed. At each physical therapy session, pain was evaluated to ensure that symptoms were not worsening. Progression of physical therapy was guided by pain exacerbation, with activity intensity increased only when pain did not worsen during or after exercise. At approximately 1-1.5 months after the initial visit (hereafter referred to as the "middle" phase), a reassessment was performed, including repeat MRI evaluation of the fracture line and BME, as well as clinical reassessment of pain using the same assessment methods as those at the initial evaluation. If there was no worsening on MRI and pain had resolved, the patient proceeded with a gradual RTS. RTS progression followed a shared principle across all participants, with gradual advancement initiated only after pain resolution and confirmation of no MRI worsening, although individual adjustments were made according to each patient’s clinical status and recovery.

**Figure 2:**
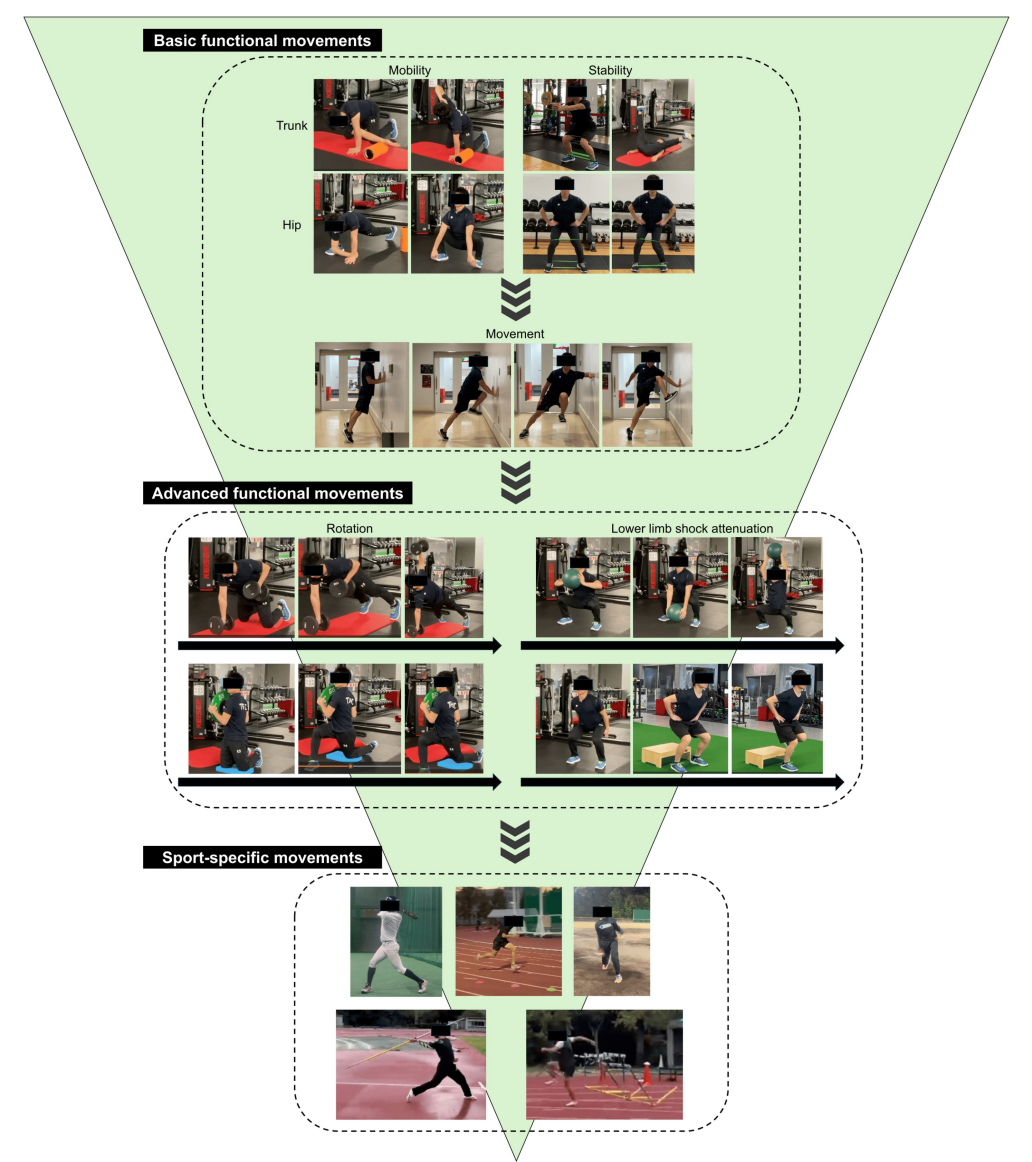
Conceptual diagram of the progressive rehabilitation approach. The diagram illustrates the stepwise progression from basic to advanced functional movements and the gradual reintroduction of sport-specific activities.

During the period from the initial visit to the middle phase, physical therapy consisted of general range-of-motion exercises, strengthening exercises, and basic functional movements based on the patient’s symptoms and injury-related movements (Figure 2). This phase focused primarily on basic functional movements aimed at improving trunk and hip mobility and stability. For the trunk, dynamic stretching for thoracic extension and rotation, as well as anti-rotation strength training of the trunk muscles, were performed. For the hips, dynamic stretching for extension and rotation and strengthening exercises targeting the gluteal muscles were implemented. Subsequently, functional movements using body weight or low resistance, including wall drills, were performed to establish hip-dominant movement patterns.

In the middle and later phases, advanced functional movements and weight training were progressively added. These exercises required higher-level whole-body motor control and focused on improving coordinated rotational movements of the trunk and hips, as well as enhancing lower-limb shock attenuation. Dynamic training, such as loaded high-speed squats using a medicine ball and landing and jumping exercises, was introduced to develop load responsiveness appropriate for sport-specific activities. Throughout these phases, sport-specific movements associated with injury-related pain were gradually reintroduced, with the aim of safely correcting and relearning movement patterns and facilitating return to competition. During supervised physical therapy sessions, exercises were generally performed for approximately 10 repetitions per set, with three to five sets, and the number of sets was adjusted based on each patient’s performance capacity and symptom response. Home exercise programs were self-managed by the patients.

Outcome assessment

Treatment outcomes were assessed in terms of changes on MRI, pain status, RTS rate, RTS period, and follow-up duration. Regarding MRI findings, the presence or absence of a fracture line was assessed at both the initial and middle phases as negative or positive. BME was assessed as negative or positive at the initial visit, and at the middle phase, it was evaluated in four categories compared to the initial MRI: complete disappearance, partial reduction, unchanged, or worsening. Pain was assessed as either negative or positive at both time points. RTS was defined, following previous studies [20], as return to the pre-injury sports level. The RTS rate was defined as the proportion of patients achieving RTS, and the RTS period was defined as the time from the initial visit to RTS. The follow-up duration was defined as the time from the initial visit to the completion of conservative treatment. Follow-up duration was determined based on clinical recovery, confirmation of return to sport, and routine outpatient follow-up, resulting in variability among participants.

## Results

The patient characteristics are summarized in Tables 1, 2. Ages ranged from 17 to 19 years. There were four males and two females. The sports included two cases of track and field (hurdles), one case of javelin throw, two cases of baseball, and one case of softball. Regarding lesion characteristics, the vertebral levels affected were L5 in two cases, L4 in three cases, and L3 in one case. One case was bilateral, and five were unilateral (right in one case and left in four cases). Five cases were classified as very early stage, and one case was classified as early stage. Etiological factors were classified into four categories: contralateral terminal-stage lesions (n=1, 16.7%), traumatic episodes (n=4, 66.7%), excessive exercise (n=1, 16.7%), and alternation in the athletic event (n=0, 0%). Among the four athletes classified as having traumatic episodes, a post hoc analysis of injury-related movements further identified two distinct patterns. Two cases (50.0%) involved external forces from the ground, such as lead leg landing during hurdling or deceleration during sprinting, and two cases (50.0%) involved hyperlateral flexion caused by imbalance during the follow-through phase of batting. Specific sport movements that provoked pain were identified in all cases.

**Table 1:** Demographic and sport-related characteristics of the study participants, including age, sex, sport, dominant side, affected vertebral level, laterality of lesions, and disease stage. R, right; L, left

Case	Age (years)	Sex	Sport	Dominant side	Affected vertebral level	Side of lesions	Stage (R/L)
1	20	Female	Softball	Throwing: R Batting: R	L4	Bilateral	Very early / Terminal
2	18	Male	Track and field (Hurdle)	Lead leg: R	L5	Left	Very early
3	17	Male	Baseball	Throwing: L Batting: L	L4	Left	Very early
4	19	Male	Track and field (Hurdle)	Lead leg: L	L5	Left	Very early
5	19	Female	Track and field (Javelin)	Throwing: R Lead leg: L	L4	Left	Very early
6	19	Male	Baseball	Throwing: R Batting: L	L3	Right	Early

**Table 2:** Etiological factors, injury-related movements, movement patterns, and sport-specific actions associated with pain onset in the study participants.

Case	Etiological factors	Injury-related movement	Injury-related movement pattern	Specific sport movements provoking pain
1	Contralateral terminal stage	-	-	Right foot contact during sprinting
2	Traumatic episode	Left lower limb landing	Reaction force from the ground	Lead leg landing during hurdling
3	Traumatic episode	Left lateral flexion in batting follow-through	Hyper-lateral flexion	Sprint deceleration phase Batting follow-through
4	Traumatic episode	Deceleration (Unknown details)	Reaction force from the ground	Sprint deceleration phase
5	Excessive exercise	-	-	Lead leg contact in javelin throw
6	Traumatic episode	Right lateral flexion in batting follow-through	Hyper lateral flexion	Batting follow-through

Treatment outcomes are summarized in Table 3. MRI improvement was observed in five cases (83.3%), and pain resolution was achieved in all six cases (100%). The RTS rate was 100%, with a median RTS period of 65.0 days (range, 54-112 days). The median follow-up duration was 109 days (range, 54-130 days), and no recurrences of low back pain were observed during the follow-up period.

**Table 3:** Treatment outcomes. BME, bone marrow edema; RTS, Return to sport; K, Kemp; E, Extension; F, Flexion; -, It was not possible to obtain MRI images at this time point.

Case	Fracture line		BME		Pain		RTS period (days)	Follow-up duration (days)
	Initial visit	Middle phase	Initial visit	Middle phase	Initial visit	Middle phase		
1	Negative	Negative	Positive	Improved	K and E	Negative	56	91
2	Negative	Negative	Positive	Unchanged	K and E	Negative	66	125
3	Negative	Negative	Positive	Improved	E	Negative	64	99
4	Negative	-	Positive	-	F	Negative	54	54
5	Negative	Negative	Positive	Negative	K and E	Negative	112	130
6	Positive	Negative	Positive	Improved	K and E	Negative	84	119

Case descriptions

Cases 5 and 6 were selected for detailed description because they demonstrated characteristic injury mechanisms and rehabilitation progressions that were particularly illustrative of the PR approach.

Case 5

**Figure 3:**
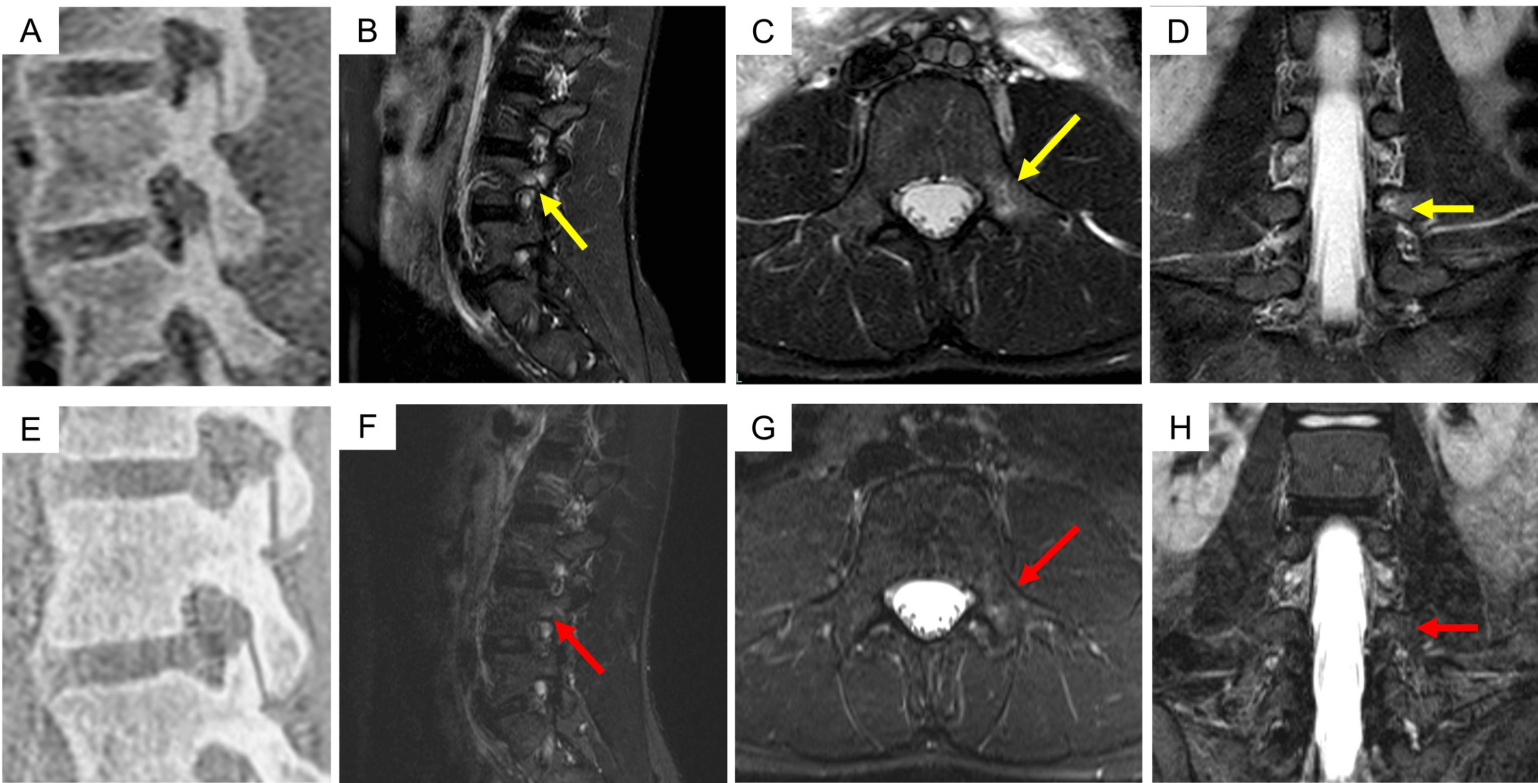
MRI images of Case 5 showing a lesion at the left L4 pars interarticularis. MRI scans obtained at the initial visit (A–D) and one month later (E–H) are presented. No fracture lines were visible on the initial MRI images; however, BME was observed (yellow arrows). At the one-month follow-up, the BME had resolved (red arrows). MRI, magnetic resonance imaging; BME, bone marrow edema; A, B, E, and F, sagittal view through the right L4 pedicle; C and G, axial view through L4; D and H, coronal view through the left L4 pedicle.

**Figure 4:**
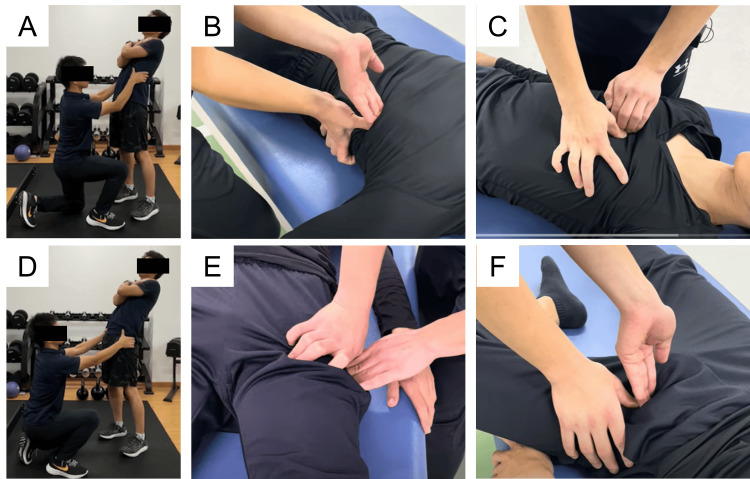
Representative components of the physical therapy intervention. During trunk extension, pain reduction was assessed by facilitating thoracic expansion (A). If pain was alleviated by this maneuver, massage was applied to the latissimus dorsi (B) and pectoralis major (C) muscles. Additionally, posterior pelvic tilt facilitation was used to evaluate pain alleviation during trunk extension (D). When effective, massage was administered to the hip flexors (E) and the inguinal region (F).

A right-handed female collegiate javelin thrower with a history of a left transverse process fracture in junior high school developed a gradual onset of left-sided lower back pain during lead leg landing after increasing her training workload at university. She presented 115 days after symptom onset and was diagnosed with very-early-stage ALS at left L4 (Figure 3). Throwing was discontinued until the middle stage, and physical therapy was initiated. Figure 4 shows part of the physical therapy. Pain improved through facilitated thoracic expansion and posterior pelvic tilt, with greater relief from thoracic correction. Therefore, thoracic expansion was prioritized, followed by posterior pelvic tilt. Basic functional movements, including hip hinge training and maintenance of a neutral spine, were also performed to improve lead leg landing mechanics (Figure 2). At the middle stage, MRI showed complete disappearance of BME, with resolution of pain. Advanced functional movements, including landing drills, were introduced, and throwing was gradually resumed. She returned to competition 112 days after the initial visit.

Case 6

**Figure 5:**
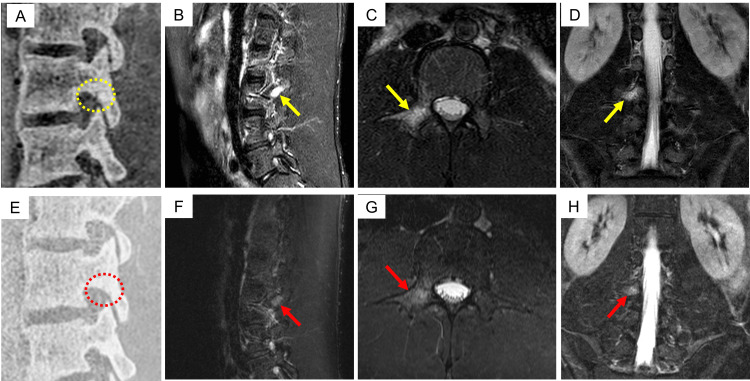
MRI images of Case 6 showing a lesion at the right L3 pars interarticularis. MRI scans obtained at the initial visit (A–D) and 1.5 months later (E–H) are presented. At the initial visit, a fracture line and bone marrow edema (BME) were observed (yellow circles and arrows). At the 1.5-month follow-up, the fracture line had disappeared, and BME was reduced (red circles and arrows). MRI, magnetic resonance imaging; BME, bone marrow edema; A, B, E, and F, sagittal view through the right L3 pedicle; C and G, axial view through L3; D and H, coronal view through the L3 pedicle.

A left-handed male collegiate baseball player developed right-sided low back pain after losing balance during a missed swing with excessive lateral trunk bending. He presented 78 days after symptom onset and was diagnosed with ALS at right L3 (Figure 5). Batting was discontinued until the middle stage, and physical therapy similar to that in Case 5 was initiated. To reduce lateral trunk bending, functional movements emphasized leading leg hip function, hip hinge and rotation training, and neutral spine control (Figure 2). At the middle stage, MRI showed complete disappearance of the fracture line, partial reduction of BME, and pain resolution. Advanced functional movements with an increased load were then performed to enhance hip-trunk coordination and whole-body stiffness. Batting was gradually resumed, and competition was permitted after he successfully completed full practice sessions. He returned to competition 84 days after the initial visit.

## Discussion

This study investigated the patient characteristics and clinical outcomes of high-level high school and collegiate athletes with ALS who underwent the PR approach. A notable feature of this study is the classification of injury-related movement patterns, specifically in cases in which the etiological factor was a traumatic episode. Additionally, reports on conservative treatment for ALS in high-level athletes remain limited [5, 6, 9], and to the best of our knowledge, no prior studies have reported an approach similar to the one used in this study. Therefore, the findings of the present study are clinically relevant.

Regarding patient characteristics, the types of sports and affected vertebral levels were consistent with those reported in previous studies [5, 6]. Regarding etiological factors, a traumatic episode was present in 66.7% of cases, which could be classified into two groups: those involving high ground reaction forces and those involving excessive lateral bending. In the high ground reaction force group (Cases 2 and 4), spondylolysis occurred on the same side as the landing leg. Single-leg landing and deceleration movements generate high ground reaction forces, causing shock attenuation through the lower limbs, particularly at the hips [21]. Failure of shock attenuation likely resulted in excessive stress on the pars interarticularis, suggesting a potential association between the landing side and the lesion side. In the excessive lateral bending group (Cases 3 and 6), the lesion occurred on the same side as the bending direction. Stress on the lumbar facet joints is reported to be highest during combined lateral bending and rotation [22], indicating that excessive lateral bending may have increased stress on the pars via the facet joints, suggesting a possible relationship between the bending and lesion sides. These findings indicate that, in cases in which a traumatic episode is the etiological factor, a detailed evaluation of the injury-related movement may help elucidate the mechanism of stress on the pars interarticularis and provide valuable guidance when designing movement retraining interventions.

Regarding the outcomes of the PR approach, the RTS rate was 100%, and the median RTS period was 65.0 days, indicating an earlier RTS period compared with prior reports that described an RTS period of approximately five months [6]. We believe that two key factors contributed to these favorable outcomes. First, all high-level athletes in this study had either a clear injury mechanism or a specific sports movement that provoked pain. By restricting, modifying, and gradually reintroducing injury-related movements, the PR approach aimed to reduce excessive stress on the pars interarticularis. These movement adjustments likely decreased shear forces by promoting more hip-dominant movement patterns and improving trunk-hip coordination, thereby creating a mechanical environment conducive to bone healing. Second, by not implementing activity restriction as a general rule, the decline in physical performance and changes in body composition reported in previous studies [10-15] were likely minimized. Furthermore, no recurrence of low back pain was observed during the follow-up period, supporting the safety of the PR approach.

Regarding the indications for the PR approach, both the athletes’ age group and their understanding of the risks related to bone union are important considerations. During the growth period, injury-related movements or pain-provoking movements may not always be identifiable, and the risk of progression to spondylolisthesis is reportedly higher [23]. In contrast, all high-level athletes included in this study demonstrated either a clear injury-related movement or a specific movement that provoked pain, which we believe contributed to effective progression of treatment and pain management. Previous studies have reported favorable bone healing with conventional conservative treatment involving activity restriction [18, 24]. Therefore, traditional conservative treatment remains the recommended approach when bone union is considered the highest priority. In contrast, Tezuka et al. [6] emphasized that for high-level athletes, pain relief may be more clinically relevant than bony union, suggesting that treatment strategies should be individualized based on the athlete’s competitive level and needs. Based on these perspectives, we propose that the PR approach is best suited for post-growth high-level athletes who fully understand the associated risks and prioritize early return to sport. Regarding disease staging, all patients in this study were in the very early or early stages. In such stages, if worsening is observed on follow-up MRI at the middle phase, the treatment strategy can be modified and shifted to conventional conservative therapy aimed at achieving bone union. However, in progressive-stage cases, further progression may lead to the terminal stage, in which bone union is difficult to achieve. Therefore, the PR approach may not be appropriate for cases in the progressive stage. Further studies are needed to clarify stage-specific indications.

This study has three limitations. First, MRI follow-up could not be continued until complete radiological resolution, such as disappearance of bone marrow edema or fracture lines, was confirmed in all patients. Instead, the absence of radiological worsening on MRI at the middle phase was used as an indicator of treatment progress. Although continued physical activity was permitted, no imaging deterioration was observed, suggesting that mechanical stress on the lumbar spine may have been reduced and that reparative processes could proceed despite ongoing activity. Second, the follow-up period was relatively short, and long-term outcomes need to be clarified in future investigations. Third, because cases of ALS in high-level athletes are relatively rare, the small sample size made the influence of prior medical history unclear. In Case 6, which demonstrated a prolonged RTS period, a history of low back problems was documented. The relationship between RTS duration and prior medical history requires further investigation. This finding also highlights the importance of obtaining a thorough clinical history during patient interviews. In addition, the PR approach was described as a conceptual, pain-guided rehabilitation framework rather than a fully standardized exercise protocol. Exercise selection, loading parameters, frequency, and progression were individualized based on clinical judgment and symptom response, and pain was assessed based on the presence or absence of symptom exacerbation rather than using a numerical scale. While this approach reflects real-world clinical practice in high-level athletes, it introduces subjectivity and limits strict reproducibility and immediate prescriptive application. Future prospective studies should aim to operationalize these principles into more standardized protocols with clearly defined exercise parameters and progression criteria. Because this study was a retrospective case series in high-level athletes, future studies should increase the sample size, compare the clinical outcomes of the PR approach with those of conventional conservative therapy, and examine differences in physical function [25, 26] between high-level athletes and individuals in the growth phase. Such investigations may help clarify the safety and applicability of the PR approach.

## Conclusions

This study investigated the patient characteristics and treatment outcomes of high-level high school and collegiate athletes with ALS who underwent the PR approach. Traumatic episodes were the most frequent etiological factor, primarily involving high ground reaction force movements or excessive lateral bending caused by a loss of balance. The PR approach achieved a 100% RTS rate and a shorter RTS period compared with previous studies. We consider appropriate candidates for the PR approach to be post-growth high-level athletes who fully understand the risks related to bone healing and aim for early RTS; however, further studies are warranted.
